# Resting‐State Functional MRI Analyses for Brain Activity Characterization: A Narrative Review of Features and Methods

**DOI:** 10.1111/ejn.70276

**Published:** 2025-10-23

**Authors:** Alejandro Amador‐Tejada, Bhanu Sharma, Ethan Danielli, Michael D. Noseworthy

**Affiliations:** ^1^ School of Biomedical Engineering McMaster University Hamilton Ontario Canada; ^2^ Imaging Research Centre, St. Joseph's Healthcare Hamilton Ontario Canada; ^3^ Electrical and Computer Engineering McMaster University Hamilton Ontario Canada; ^4^ Child Health and Exercise Medicine Program McMaster University Hamilton Ontario Canada; ^5^ Department of Kinesiology McMaster University Hamilton Ontario Canada; ^6^ Department of Medical Imaging McMaster University Hamilton Ontario Canada

**Keywords:** blood‐oxygen‐level‐dependent (BOLD) signal, functional magnetic resonance imaging (fMRI), global brain activity, local brain activity, low‐frequency fluctuations, neuroimaging, resting state

## Abstract

Resting‐state fMRI (rsfMRI) is a widely used neuroimaging technique that measures spontaneous fluctuations in brain activity in the absence of specific external cognitive, motor, emotional, and sensory tasks or stimuli, based on the blood‐oxygen‐level‐dependent (BOLD) signal. Functional connectivity (FC) is a popular rsfMRI analysis examining BOLD signal correlations between brain regions. Nevertheless, there are alternative analyses that provide different but collectively informative characteristics of the BOLD signal and, thus, brain activity. This narrative review aimed to provide a comprehensive conceptual, mathematical, and significance investigation of common rsfMRI analyses in addition to FC. To achieve this, a narrative review was conducted on studies using the most common rsfMRI analysis to investigate global and local brain activity. Five rsfMRI analyses were described, summarizing the common initial steps of rsfMRI data processing and explaining the main characteristics and how each metric is calculated. The rsfMRI analyses described are (1) FC, reflecting BOLD global connectivity; (2) the amplitude of low‐frequency fluctuations (ALFF) and fractional ALFF (fALFF), representing the intensity of the BOLD signal; (3) regional homogeneity (ReHo), which reflects BOLD local connectivity; (4) Hurst exponent (H), depicting autocorrelation of the BOLD signal; and (5) entropy, depicting the BOLD signal predictability. As rsfMRI is a vital tool for exploring brain function, selecting an analysis that aligns with the research question is essential. This review offers an initial catalog of standard rsfMRI analyses, highlighting their key features, concepts, and considerations to support informed decisions by researchers and clinicians.

## Graphical abstract

Functional connectivity is a popular resting‐state functional MRI analysis. Nevertheless, there are alternative approaches that can be used to characterize the BOLD signal differently, which allow for indirect characterization of brain activity, focusing on regional connectivity, intensity, and complexity. This review offers an initial core set of standard analyses, highlighting their key features, concepts, and considerations to support informed decisions by researchers and clinicians.

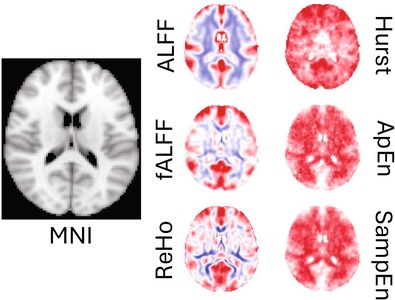

**Significance Statement**

Resting‐state functional MRI (rsfMRI) is a widely used technique for studying brain function in the absence of external stimuli. Several analyses are available to study different aspects of brain function, yet no recent review has consolidated commonly used rsfMRI analyses. Given the popularity of rsfMRI in both research and clinical settings, a clear overview is essential to guide appropriate method selection, calculation, and context for interpretation. Therefore, this review aims to provide a comprehensive conceptual, mathematical, and significance assessment of five rsfMRI analyses, representing global and local brain connectivity and local intensity, predictability, and autocorrelation of brain activity and function.

**Abbreviations**
ALFF: amplitude of low‐frequency fluctuations
ApEn: approximate entropy
BOLD: blood‐oxygen‐level‐dependent
fALFF: fractional ALFF
fBm: fractional Brownian motion
FC: functional connectivity
FD: fractal dimension
fGn: fractal Gaussian noise
fMRI: functional magnetic resonance imaging
H: Hurst exponent
ICA: independent component analysis
KCC: Kendall's coefficient of concordance
LFF: low‐frequency fluctuations
MB: multiband
PSD: power spectral density
ReHo: regional homogeneity
ROI: region of interest
rsfMRI: resting‐state fMRI
SampEn: sample entropy

## 1 Introduction

Functional magnetic resonance imaging (fMRI) is a popular, noninvasive MRI technique introduced in 1990 when signal changes in $T_{2}^{*}$‐weighted images of rat brains were attributed to changes in blood flow and oxygenation (Ogawa et al. 1990). Most fMRI methods probe the blood‐oxygen‐level‐dependent (BOLD) signal, an indirect method to assess neuronal activity (Uludag et al. 2015). The BOLD signal is based on the complex neurovascular interaction between regional changes in cerebral perfusion, blood volume, and metabolism that are a result of locally increased neuronal activity and metabolism (Arthurs and Boniface 2002; Logothetis 2008). The BOLD signal changes due to temporal distortions in the local magnetic field caused by a change in the ratio of oxy‐hemoglobin (oxyHb; diamagnetic) to deoxyHb (paramagnetic), which results from neuronal activation (Buxton 2013; Raichle 2001). For instance, an active brain area demands higher oxygen concentrations for metabolism and thus causes an increase in localized blood flow and more oxyHb being delivered to the active region. The relative decrease of deoxyHb compared to oxyHb reduces the local magnetic field distortions, increasing the BOLD signal (Hyder et al. 2001).

**FIGURE 1:**
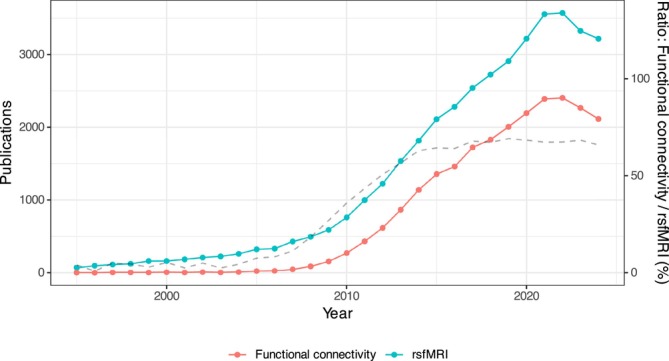
Plot illustrating the number of publications from 1995 to 2024 related to all types of rsfMRI analyses (blue). The number of publications specifically focused on FC is shown in red. The grey line represents the percentage of rsfMRI publications dedicated to FC analysis, calculated as $\frac{\#\text{functional connectivity publications}}{\#\text{rsfMRI publications}}\times 100\left(\%\right)$. The keywords to generate this plot are shown in Table 1.

There are two primary approaches to acquiring fMRI data, namely, resting‐state fMRI (rsfMRI), wherein the brain activity is measured in the absence of any specific stimuli, and task‐based fMRI, where the BOLD signal is measured in response to cognitive, motor, emotional, and sensory tasks or stimuli. rsfMRI was first introduced in 1995 (Biswal et al. 1995) as a technique to study the spontaneous low‐frequency fluctuations (LFF) (< 0.1 Hz) of the BOLD signal in the brain at rest, without any stimulus or task. Even though the definition of “resting state” is nuanced, any fMRI scan completed without a task or stimulus is considered rsfMRI. It became an essential approach to studying brain function because of its reproducibility and flexibility, as it does not require complicated experimental paradigms, broadening the eligibility criteria to include participants with neurological and psychiatric conditions (Gohel et al. 2019; Lee et al. 2013; Smitha et al. 2017). Moreover, compared to task‐based fMRI, the “resting state” of the brain accounts for approximately 60%–80% of its total metabolic demand (Mastrovito 2013; Smitha et al. 2017). As such, using rsfMRI to indirectly measure core brain activity captures a large portion of the brain's physiological activity and health. The popularity of rsfMRI increased steadily until 2022, after which it declined slightly (Figure 1).

Continued rsfMRI research showed that physiologically relevant spontaneous fluctuations were synchronized across brain regions (Beckmann et al. 2005; Biswal et al. 1995). This brain BOLD signal synchrony suggests that anatomical regions are working “together” and are functionally connected, which, at rest, is considered to reflect a state of self‐consciousness, unconstrained mental activity, and intrinsic spontaneous neuronal fluctuation (Birn 2012; Cordes et al. 2000; Gruber et al. 2018; Haughton and Biswal 1998). The rsfMRI analysis to study functional connectivity (FC) is widely known as resting‐state FC fMRI and has gained widespread popularity in the scientific community (Chen et al. 2020; Rosazza and Minati 2011). Moreover, the number of FC publications exhibited a similar trend to rsfMRI publications, positioning itself as the leading rsfMRI technique (Figure 1). FC has become the predominant rsfMRI analysis in recent years (Logothetis 2008; S. M. Smith et al. 2013; van den Heuvel and Hulshoff Pol 2010; J. Yang et al. 2020), possibly due to its simplicity and robust reproducibility. In its basic form, it measures between‐voxel BOLD signal synchronicity through Pearson's correlation coefficient (Bijsterbosch et al. 2017; Mohanty et al. 2020).

Nevertheless, the BOLD signal time series can provide distinct and complementary information about brain function and organization. These characteristics, although less commonly utilized, are not limited to global temporal synchronization as measured by FC. For instance, the amplitude of LFF (ALFF) and fractional ALFF (fALFF) indirectly measure regional patterns of neuronal activity levels by capturing BOLD signal power within low‐frequency oscillations (H. Yang et al. 2007; Zou et al. 2008). Alternatively, regional homogeneity (ReHo) assesses BOLD signal FC using Kendall's coefficient of concordance (KCC), quantifying the regional coherence of areas within the brain (Zang et al. 2004). In addition, the Hurst exponent (H) evaluates BOLD signal complexity by looking at self‐similarity, autocorrelation, and fractal properties (Ciuciu, Varoquaux, et al. 2012). Entropy is another example representing BOLD signal complexity, evaluating its predictability over time (Saxe et al. 2018). While FC remains the most popular, these several other analyses allow for investigation of other BOLD signal characteristics that can be indicative of brain health (Cole et al. 2010; Lee et al. 2013). Whereas FC explores global brain networks and ReHo local brain networks, these alternative approaches represent additional features of voxel‐wise brain activity in grey matter.

Thus, a framework that integrates these diverse characteristics alongside FC is essential to fully understand the multifaceted nature of the BOLD signal and how it can be used in different contexts. Far more information can be drawn from the BOLD signal than correlations calculated with FC. This narrative review aims to provide an updated and comprehensive review of these five established rsfMRI analyses (viz., FC, ALFF and fALFF, ReHo, H, and entropy). To ensure clear interpretation and practical applicability with minimal analytic complexity, we focus on a compact set of rsfMRI metrics with standardized pipelines and strong reliability. Therefore, other, more complex metrics, such as effective connectivity (Seth et al. 2015; Sharaev et al. 2016) or Lempel–Ziv complexity (Shumbayawonda et al. 2018) were not considered. With rsfMRI analysis methods advancing rapidly, a timely review is needed. This work synthesizes the core principles, strengths, and limitations of key rsfMRI approaches, providing researchers with a reference point for selecting the most appropriate metric for their objectives and research context. This review paper aims to provide an overview of rsfMRI analyses, examining beyond traditional techniques, such as FC, while referencing available software to facilitate and accelerate the adoption of more advanced rsfMRI analysis.

This is a narrative review following relevant guidelines and recommendations (Ferrari 2015; Green et al. 2006). Searches were performed using PubMed, Scopus, and Google Scholar. All searches were limited to humans, with studies published in English and no limitations on the publication year. Inclusion criteria included rsfMRI‐related publications, both sexes, with healthy and disease populations (e.g., attention‐deficit/hyperactivity disorder [ADHD], schizophrenia, Alzheimer's disease [AD], and major depressive disorder [MDD]). The inclusion of studies involving clinical populations was intentional, since some rsfMRI analyses were originally conceived and validated in clinical contexts, whereas others first emerged from basic science work. For this reason, the criteria allowed both healthy and clinical populations, while the overall focus of the review remains methodological rather than clinical. All studies were considered for inclusion in this review, including narrative reviews, systematic reviews, and original research articles (OSF preregistration osf.io/psxnz) (Amador‐Tejada et al. 2024).

The content for each rsfMRI analysis included in this review covers a conceptual overview explaining what is being measured, its significance, and limitations, as well as a mathematical overview describing how the metric is computed. Analysis‐specific data preprocessing steps are mentioned, as required. In addition, a summary of search term keywords (Table 1), main characteristics (Table 2), and significance and interpretation (Table 3) is shown for each rsfMRI analysis approach.

**TABLE 1:** Keywords used for rsfMRI‐related research papers and for each rsfMRI analysis. For each analysis, the search query consisted of the keywords for rsfMRI plus the specific keywords of each analysis.

	Keywords
rsfMRI	(“functional Magnetic Resonance Imaging” OR “fMRI” OR “functional MRI”) AND (“resting‐state” OR “resting state” OR “rest” OR “resting” OR “rsfMRI” OR “rs‐fMRI” OR “spontaneous fluctuations” OR “task‐free”)
Functional connectivity (FC)	“fc” OR “fc*fmri” OR “functional connectivity” OR “intrinsic connectivity”
Amplitude of low‐frequency fluctuations (ALFF) and fractional ALFF (fALFF)	(“alff” OR “amplitude of low*frequency fluctuation*”) OR (“falff” OR “fractional amplitude of low*frequency fluctuation*”)
Regional homogeneity (ReHo)	“regional homogeneity” OR “reho”
Hurst exponent (H)	“hurst” OR “hurst exponent” OR “fractal dimension” OR “scale‐free”
Entropy	“entropy”

**TABLE 2:** Summary of rsfMRI analysis, showing its domain (time and frequency), whether the rsfMRI analysis is computed to indirectly depict local or global functional properties of the brain, and any available software.

rsfMRI analysis	Domain	Spatial features	Available software
Functional connectivity (FC)	Time	Global networks	CONN: Functional Connectivity Toolbox (Whitfield‐Gabrieli and Nieto‐Castanon 2012) Data Processing Assistant for Resting‐State fMRI (DPARSF) (Chao‐Gan and Yu‐Feng 2010)
Amplitude of low‐frequency fluctuations (ALFF) and fractional ALFF (fALFF)	Frequency	Local brain activity (i.e., voxel‐wise)	Resting‐state fMRI Data Analysis Toolkit (REST) (X. W. Song et al. 2011) CONN: Functional Connectivity Toolbox (Whitfield‐Gabrieli and Nieto‐Castanon 2012)
Regional homogeneity (ReHo)	Time	Local networks	Resting‐state fMRI Data Analysis Toolkit (REST) (X. W. Song et al. 2011) Data Processing Assistant for Resting‐State fMRI (DPARSF) (Chao‐Gan and Yu‐Feng 2010)
Hurst exponent (H)	Frequency	Local brain activity (i.e., voxel‐wise)	Github python‐based scripts (Campbell et al. 2021) Python PyPI package—Hurst (Mottl 2019)
Entropy	Time	Local brain activity (i.e., voxel‐wise)	Brain Entropy Mapping Toolbox (BENtbx) (Z. Wang et al. 2014)

**TABLE 3:** Summary of the takeaway fundamentals of each rsfMRI analysis. To note that the theoretical ranges do not reflect image normalization as typically performed in ALFF, fALFF, and ReHo, as shown in Figures 3 and 4.

rsfMRI analysis	Significance	Theoretical range	Interpretation
Functional connectivity (FC)	Representation of the functional synchrony of the BOLD signal across the brain (Biswal et al. 2010)	$\left[-1,1\right]$	Values within $\left(0,1\right]$ indicate concordance, while values within $\left[-1,0\right)$ suggest anticorrelation between the BOLD signal and the reference signal. An FC value of 0 indicates no correlation.
Amplitude of low‐frequency fluctuations (ALFF) and fractional ALFF (fALFF)	Reflects the intensity of the BOLD signal (Zou et al. 2008), suggestive of the magnitude of neuronal activity (Y. F. Zang et al. 2007)	ALFF $\left[0,\infty \right)$ fALFF $\left[0,1\right]$	Increased values reflect higher power in the BOLD signal, whereas decreased values indicate lower power.
Regional homogeneity (ReHo)	Represents the temporal correlation between a voxel and its neighbors, that is, “local” FC (Golestani et al. 2017; Q. H. Zuo et al. 2013)	$\left[0,1\right]$	Increased values propose higher synchrony between a given voxel and its neighbors of the BOLD signal, while decreased values indicate lower synchrony.
Hurst exponent (H)	Reflects the self‐similarity of the BOLD signal, suggestive of fractal complexity of the brain (Ciuciu, Varoquaux, et al. 2012; Dong et al. 2018)	$\left[0,1\right]$	Values within $\left[0,0.5\right)$ suggest a more complex signal, while values within $\left(0.5,1\right]$ suggest a less complex BOLD signal. A Hurst value of 0.5 is indicative of an uncorrelated noise (fGn) or random walk pattern (fBm).
Entropy	Shows how “predictable” is the BOLD signal (Saxe et al. 2018; Z. Wang 2021).	$\left[0,\infty \right)$	Increased values translate to less predictability, while decreased values depicts to more predictability of the BOLD signal.

## 2 Standard Preprocessing for rsfMRI Data

It is essential to perform standard preprocessing of the raw rsfMRI data to denoise and prepare it for subsequent analysis. Preprocessing should remain consistent across subjects in a study or a given analysis. Standard rsfMRI preprocessing typically includes (1) deletion (or simply to not save these data) of the first 5–10 functional volumes to allow for magnetization equilibrium; (2) slice‐timing correction to correct for differences in acquisition time between slices for each acquired volume; (3) motion correction to correct for head movements during the scanning session; (4) spatial smoothing to increase signal‐to‐noise ratio, performed with a Gaussian kernel set to a full‐width at half‐maximum (FWHM) typically within 4–8 mm (Mikl et al. 2008); (5) 4D global normalization to mitigate interscan variability; (6) brain extraction to remove all nonbrain tissues (i.e., skull, muscle, skin) to isolate the brain for subsequent computations; and (7) registration of the participant's brain to standard brain space (e.g., MNI, N27, and Talairach space) to reduce intersubject variability and to allow group‐wise statistics (Biswal et al. 2010). In addition, if available, eddy current correction can be performed to remove magnetic field warping caused by magnetic field gradient‐induced eddy currents. Moreover, additional preprocessing steps are required as each analysis focuses on different aspects of the BOLD signal, which is described for each (below).

Available software typically used to perform this routine rsfMRI preprocessing includes FSL (Jenkinson et al. 2012; S. M. Smith, Jenkinson, et al. 2004; Woolrich et al. 2009), AFNI (Cox 1996; Cox and Hyde 1997), Resting‐state fMRI Data Analysis Toolkit (REST) (X. W. Song et al. 2011), CONN: Functional Connectivity Toolbox (Whitfield‐Gabrieli and Nieto‐Castanon 2012), fMRIPrep (Esteban et al. 2019), SPM (Penny et al. 2011), Configurable Pipeline for the Analysis of Connectomes (C‐PAC) (Craddock et al. 2013), Data Processing Assistant for Resting‐State fMRI (DPARSF) (Chao‐Gan and Yu‐Feng 2010), and many others.

## 3 rsfMRI Analyses

### 3.1 FC

FC is defined as the temporal correlation between spontaneous BOLD signals in spatially distinct brain regions (Biswal et al. 1995; Friston et al. 1993). Resting‐state FC MRI is a class of analyses used to study connectivity on a whole‐brain scale with respect to correlated, synchronized functional activity between individual brain regions or between clusters of brain regions (Bijsterbosch et al. 2017; Fox and Raichle 2007). FC aims to identify brain regions whose BOLD time series are correlated throughout the rsfMRI scan and measure the direction (i.e., positive or negative) and strength of their correlation. Theoretically, if two brain regions show similarities in their BOLD signal over time, they are “functionally” connected (Bijsterbosch et al. 2017). Moreover, FC captures correlations between spatially distinct BOLD signals, indicating that functional networks are partly constrained by structural architecture (Greicius et al. 2009; Honey et al. 2009; Z. Wang et al. 2013).

There are three principal approaches for FC: model‐driven (seed‐based), data‐driven (independent component analysis [ICA]), and graph theory (Lee et al. 2013; Margulies, Bottger, et al. 2010). Seed‐based FC (seed‐to‐voxel and region of interest [ROI]‐to‐ROI) remains among the easiest and most common ways to examine FC (Biswal et al. 1995; van den Heuvel and Hulshoff Pol 2010). In seed‐to‐voxel analysis, the bivariate correlation coefficient between a predefined seed (e.g., anatomically defined based on an atlas or manually created ROIs) and all other voxels in the brain is computed, producing a correlation map whose values range from −1 to 1 and reflect the level of relationship between the seed (i.e., the “reference” BOLD signal) and each voxel in the brain across the time series (Fox and Raichle 2007; Joel et al. 2011; Rogers et al. 2007). In ROI‐to‐ROI analysis, the brain is parcellated into predefined ROIs (typically using a defined neuroanatomical atlas), the mean BOLD time series is extracted from each, and pairwise correlations are computed to form a symmetric connectivity matrix. This reduces data dimensionality compared to seed‐to‐voxel and is well suited for both network‐level and graph‐theoretical studies (Cole et al. 2010; Fox and Raichle 2007; Poldrack 2007).

More sophisticated ICA methods model BOLD signals as linear mixtures of different sources (e.g., physiologically relevant signals and residual noise or artifacts) and decompose them into statistically independent spatial components without a priori information (Beckmann and Smith 2004; Biswal and Ulmer 1999; Calhoun, Adali, et al. 2001; Griffanti et al. 2014; Kiviniemi et al. 2003; van de Ven et al. 2004). This decomposition represents temporal and spatial components and how these signals evolve over time (Bijsterbosch et al. 2017). Both seed‐based and ICA methods can define reproducible functionally connected spatial regions known as resting‐state networks (RSNs), such as the default mode network (DMN), salience network, and central executive network (CEN), among others (Seitzman et al. 2019). Lastly, graph theory is a mathematical framework that models the brain as a network, where nodes represent defined anatomical brain regions, and edges depict a measurement of association between each pair of nodes (Bijsterbosch et al. 2017; Bullmore and Sporns 2009; Margulies, Bottger, et al. 2010). Graph theory measures properties of interest from nodes and edges of modeled brain networks, including node degree (the number of connections a node has), path length (the shortest path between a pair of nodes), clustering coefficient (the degree to which connected nodes in the network are clustered), and modularity (the extent to which the network can be subdivided into smaller communities) (Bullmore and Sporns 2009; van den Heuvel and Hulshoff Pol 2010). Several excellent and comprehensive reviews on graph theory in neuroimaging are available, and those works provide a more detailed explanation of the topic (Bassett and Bullmore 2006; Bullmore and Sporns 2009; Bullmore and Bassett 2011).

Along with standard rsfMRI preprocessing steps (Section 2 Standard Preprocessing for rsfMRI Data), these additional preprocessing steps are recommended: (1) removal of nuisance signals (white matter [WM], cerebrospinal fluid [CSF], motion parameters, and global signal), (2) band‐pass filter within the range of 0.01–0.08 or 0.01–0.1 Hz (Fox and Raichle 2007), and (3) linear detrending (Bijsterbosch et al. 2017; Biswal et al. 2010; Van Dijk et al. 2010). For data‐driven approaches such as ICA, only a high‐pass filter of 0.01 Hz is used (Biswal et al. 2010). It is worth noting that, while global signal regression reduces global and motion‐related noise (Keller et al. 2013), it is a controversial step, as it can introduce spurious negative correlations and distort group differences (Gotts et al. 2013). Furthermore, because the global signal reflects neural and physiological processes, regressing it risks discarding meaningful information (Li et al. 2019; T. T. Liu et al. 2017). These trade‐offs have led to no consensus on its use as of date (Murphy and Fox 2017).

FC is a global network analysis. For a seed‐based analysis, Pearson's correlation coefficient (*r*) between a “reference” signal and every voxel in the rsfMRI preprocessed brain data is calculated as

$$r_{\text{reference},i^{th}\;\text{voxel}}=\frac{\mathrm{cov}\left(\text{reference},i^{th}\;\text{voxel}\right)\;}{\sigma_{\text{reference}}\;\sigma_{i^{th}\;\text{voxel}}},$$

where

- The reference signal is the mean of a predefined ROI.
- cov is the covariance between the reference BOLD signal and the BOLD signal from the $i^{th}$ voxel, that is, $\mathrm{cov}\left(X,Y\right)=\frac{\sum_{i=1}^{n}\left(X_{i}-\bar{X}\right)\left(Y_{i}-\bar{Y}\right)}{n}$.
- σ is the standard deviation of the reference BOLD signal or the BOLD signal from the $i^{th}$ voxel.

Correlation maps are typically transformed to *Z* values using Fisher's *r*‐to‐*z* transformation (Fox and Raichle 2007; Fox et al. 2005; C. G. Yan, Cheung, et al. 2013).

### 3.2 ALFF and fALFF

Physiologically relevant LFF at rest were first studied in the motor cortex, with WM exhibiting about a 60% lower LFF amplitude relative to grey matter (Biswal et al. 1995). Later, it was suggested that LFF (< 0.08 Hz) predominantly represent the FC across the brain (Cordes et al. 2001). Altogether, this led to the BOLD analysis known as the ALFF. ALFF measures the BOLD signal spectral power over a specific frequency range, typically within the LFF range (e.g., 0.01–0.08 Hz) (Zang et al. 2007). Power represents the overall contribution of a frequency component to the BOLD signal; therefore, ALFF measures the contribution of the LFF to the overall BOLD signal. Studies have suggested that ALFF may be used as a potential biomarker to assess the intensity of spontaneous neuronal activity (H. Yang et al. 2007; Zang et al. 2007; Zou et al. 2008) and to characterize the BOLD signal fluctuations attributable to the baseline activity of the resting brain (Fox and Raichle 2007).

ALFF is susceptible to physiological noise, such as cardiac and respiratory cycles occurring in frequencies above 0.1 Hz (Cordes et al. 2001), that produce irregular values compared to the global mean, particularly in voxels containing or adjacent to blood vessels and ventricles, and adjacent voxels (Zou et al. 2008; Zuo et al. 2010). Zou et al. (2008) introduced an improved method to effectively account for these types of physiological noise, the fALFF index. fALFF is a slight modification of ALFF, where the power of the LFF (0.01–0.08 Hz) is normalized by the power in the entire frequency range (0.01–0.25 Hz). The upper‐bound frequency is capped by the repetition time (TR). With a typical TR of 2 s (Bollmann and Barth 2021; Raimondo et al. 2021), the Nyquist theorem dictates that the maximum detectable frequency is 0.25 Hz, ensuring adequate signal sampling. Furthermore, the minimum lower‐bound frequency depends on the number of time points: $f_{\min}=\frac{1}{\text{scan}\;\text{duration}}=\frac{1}{\mathrm{TR}\times \text{time}\;\text{points}}$, yet, for typical rsfMRI scans, this theoretical lower bound is well below 0.01 Hz. In practice, however, most studies set the lower cutoff at 0.01 Hz to suppress extremely slow fluctuations arising from scanner drift, hardware instabilities, and nonphysiological processes, which can dominate these ultralow frequencies and mask neural signals (A. M. Smith et al. 1999; L. Yan et al. 2009).

In addition to standard rsfMRI preprocessing (Section 2 Standard Preprocessing for rsfMRI Data), (1) linear detrending and (2) band‐pass filtering within the range of 0.01–0.08 Hz are applied (H. Yang et al. 2007; Zang et al. 2007; Zou et al. 2008). ALFF and fALFF maps are further transformed to Z scores for each dataset, excluding the background and other tissues outside the brain. Of note is that the frequency range occasionally differs as some authors use the 0.01‐ to 0.1‐Hz range (Whitfield‐Gabrieli and Nieto‐Castanon 2012; Zuo et al. 2010). The preprocessing for fALFF is similar, except that the rsfMRI dataset is band‐pass filtered within 0.01–0.25 Hz (Zou et al. 2008).

ALFF is a local brain activity (i.e., voxel‐wise) measurement computed as the root mean square of the power spectrum of the BOLD signal within a specific frequency range (Zuo et al. 2010). ALFF is calculated as

$$\text{ALFF}=\sqrt{\sum_{\mathrm{k\epsilon}\left[0.01,\;0.08\right]\;\mathrm{Hz}}\frac{{\left|X\left[k\right]\right|}^{2}}{N}},$$

where

- $X\left[k\right]$ is the discrete Fourier transform (DFT) of the BOLD signal for the $k^{th}$ frequency
- N is the number of time points in the BOLD signal

Meanwhile, fALFF is computed as

$$\text{fALFF}=\frac{\text{ALFF}}{\sqrt{\sum_{\mathrm{k\epsilon}\left[0.01,\;0.25\right]\;\mathrm{Hz}}\frac{{\left|X\left[k\right]\right|}^{2}}{N}}},$$

where the denominator expresses the power of the BOLD signal over the entire frequency range (i.e., 0.01–0.25 Hz).

### 3.3 ReHo

The BOLD signal is not homogeneous across the brain, as it can show regional variability (Duann et al. 2002) that model‐driven approaches cannot differentiate (Zang et al. 2004). ReHo was developed as a model‐free analysis to understand these regional changes (Y. He et al. 2007; Zang et al. 2004). ReHo is a nonparametric rsfMRI analysis based on KCC, which characterizes the regional functional coherence of a given voxel and its surrounding neighbors (Long et al. 2008; C. G. Yan, Cheung, et al. 2013; Zang et al. 2004; Zuo and Xing 2014). ReHo is based on the assumption that functional coherence is more likely to happen in clusters rather than individual voxels (Y. Liu et al. 2008).

ReHo measurements are within the range [0, 1], where values closer to 1 reflect increased synchrony of the target voxel and its neighborhood, indicative of higher centrality and increased regional neuronal activity (Deng et al. 2022; Lv et al. 2018). ReHo is a robust analysis with high test–retest reliability (Zuo and Xing 2014) and little susceptibility to noise and outliers (Q. H. Zuo et al. 2013). Nevertheless, ReHo measurements can be influenced by (1) the spatial smoothing kernel size during preprocessing and (2) the number of neighbors to include in the KCC calculation (Zang et al. 2004; Q. H. Zuo et al. 2013). Furthermore, ReHo provides information on the local synchrony of isolated clusters but omits the global brain connectivity, as studied in FC. A known limitation of ReHo is that the biological basis is unclear, especially when partitioning the BOLD signal by frequency bands (X. Song et al. 2014).

In addition to standard rsfMRI preprocessing (Section 2 Standard Preprocessing for rsfMRI Data), (1) linear detrending and (2) band‐pass filtering within the range of 0.01–0.1 Hz are suggested. ReHo maps are further transformed to Z scores for each dataset, excluding the background and other tissues outside the brain (X. W. Song et al. 2011; C. G. Yan, Craddock, et al. 2013; Yu et al. 2013; Zang et al. 2004; Q. H. Zuo et al. 2013).

ReHo is a local network calculation where the KCC is computed for each voxel and its 26 voxel neighbors (Y. He et al. 2007; X. W. Song et al. 2011; Zang et al. 2004; Q. H. Zuo et al. 2013):

$$W=\frac{\Sigma_{i}{\left(R_{i}\right)}^{2}-n{\left(\frac{K\left(n+1\right)}{2}\right)}^{2}}{\frac{1}{12}K^{2}\left(n^{3}-n\right)}$$

where

- $R_{i}$ is the sum rank of the $i^{th}$time point
- K is the number of clusters, counting the specific voxel and all its neighbors, typically *K* = 27 (26 neighbors plus the specific voxel itself). Zang et al. (2004) empirically compared cluster sizes of 7, 19, and 27 voxels and found that the 27‐voxel neighborhood best balances sensitivity to local functional synchrony with preservation of spatial specificity, making it the smallest three‐dimensional neighborhood that effectively captures immediate spatial context without oversmoothing (Y. He et al. 2007; D. Liu et al. 2010; C. G. Yan, Craddock, et al. 2013; Zang et al. 2004).
- n is the number of ranks, that is, the number of time points acquired in the rsfMRI data (Zang et al. 2004).

### 3.4 H

The H and fractal dimension (FD) are a family of fractal analyses based on chaos theory that represent the complexity of time domain signals (Eke, Herman, Bassingthwaighte, et al. 2000; Eke, Herman, Kocsis, et al. 2002; Mandelbrot and Van Ness 1968) by unveiling complex or chaotic temporal dynamics in signals that appear to be random (Eke, Herman, Bassingthwaighte, et al. 2000). Fractals are typically conceived as complex geometrical shapes that, when split into parts, individually represent a reduced‐scale copy of the initial shape. Time domain signals with fractal properties often show three main properties. The first is self‐affinity, or whether smaller components of the time domain signal, scaled by a factor *r*, show comparable patterns (Campbell and Weber 2022; Eke, Herman, Kocsis, et al. 2002). Second is autocorrelation, or whether preceding values influence the values of the signal at a particular time, or whether they are short or long‐range (Dona, Hall, et al. 2017). Finally, the third is power‐law behavior, that is, the power spectra in the frequency domain (*f*) will show a 1/*f* pattern (Ciuciu, Abry, et al. 2014; Dona, Hall, et al. 2017).

BOLD signals exhibit statistical fractal properties. Their self‐affinity and autocorrelation properties are characterized by the H, which measures the ability of the BOLD signal within a set time frame to predict future BOLD signal characteristics, and FD, which measures the complexity of the frequencies contributing to the BOLD signal (Ciuciu, Abry, et al. 2014; Ciuciu, Varoquaux, et al. 2012; Eke, Herman, Sanganahalli, et al. 2012; B. J. He 2011; Zarahn et al. 1997). There are two primary families into which fractal signals are classified: fractal Gaussian noise (fGn) or fractional Brownian motion (fBm). fGn are stationary signals with constant variance, while fBm are nonstationary signals with the variance changing over time (Delignieres et al. 2005; Eke, Herman, Bassingthwaighte, et al. 2000). Both fractal signals are characterized by H. However, examining which family the signal corresponds to is crucial, as the methods to estimate H will differ (Eke, Herman, Bassingthwaighte, et al. 2000).

H values exist within the range [0, 1]. Values within 0 < H < 0.5 reflect high complexity, anticorrelation, and short memory (i.e., the BOLD signal will look chaotic, where a high value will be followed by a low value, or vice versa, and the signal will oscillate around the mean). Values within 0.5 < H < 1 show low complexity, correlation, and long memory (i.e., the BOLD signal will show a tendency, where a high value will be followed by another high value, and vice versa). Finally, H = 0.5 exhibits a random walk pattern for fBm signals or uncorrelated noise for fGn signals (Neudorf et al. 2020; Omidvarnia et al. 2022). FD values lie within the range [1, 2], where values closer to 1 indicate smoother (less complex) dynamics and values nearer to 2 reflect greater signal complexity (Bullmore et al. 2001; Eke, Herman, Bassingthwaighte, et al. 2000).

Although there are different approaches to computing H, frequency‐based (e.g., power spectral density, PSD) and time‐based (e.g., rescaled range, R/S) analyses are popular methods, as they have shown robust results for the BOLD signal analyses (Campbell and Weber 2022; Dong et al. 2018). In addition to standard rsfMRI preprocessing (Section 2 Standard Preprocessing for rsfMRI Data), high‐pass temporal filtering (0.01 Hz) is typically applied (Campbell and Weber 2022; Dong et al. 2018; von Wegner et al. 2018).

H is a local brain activity (i.e., voxel‐wise) method where the steps to compute it, based on the PSD method, are as follows:

1 The BOLD PSD is calculated. The signal is considered to show fractal properties if the PSD shows an inverse power‐law behavior:
$$|A{\left(f\right)}^{2}|\propto cf^{-\beta },$$

○ A is the amplitude of the PSD.
○ f is the $k^{th}$ frequency.
○ c is a proportionality constant.

2 The PSD is transformed into a log–log scale plot, where β is computed as the slope of a line fitting the PSD (Campbell and Weber 2022).
3 If β<1, then H is computed as $H=\frac{\beta +1}{2}$; if β>1, then H is $H=\frac{\beta -1}{2}$ (Campbell and Weber 2022; Eke, Herman, Bassingthwaighte, et al. 2000).
4 H and FD are closely related by FD+H=n+1, where n is the space dimension (Dona, Hall, et al. 2017; Dona, Noseworthy, et al. 2017; Eke, Herman, Bassingthwaighte, et al. 2000). As the BOLD signal is a 1D time domain signal, n=1 and thus FD+H=2.

where

Other methods such as scaled windowed variance, dispersional analysis, detrended fluctuation analysis, and rescaled range analysis are also available (Eke, Herman, Bassingthwaighte, et al. 2000; Eke, Herman, Sanganahalli, et al. 2012). The selection relies on the fractal classification (fGn vs. fBm) and expected H range (Delignieres et al. 2005).

### 3.5 Entropy

Entropy analyses are used to indirectly study the complexity of a time series (Pincus 1991; Richman and Moorman 2000). The concept of entropy in the context of a time series refers to the rate of “new” information generated over time (Richman and Moorman 2000), the multiple ways a system can be configured (Saxe et al. 2018), and the regularity of a system (Z. Wang et al. 2014). The human brain constantly receives, processes, and responds to sensory input, which creates neuronal pathways and brain states related to learning and memory (C. Y. Liu et al. 2013; Saxe et al. 2018; Singer 2009). The ability of the brain to switch between states in response to stimuli is closely related to its entropy, calculated by the predictability of the BOLD signal (Saxe et al. 2018; Z. Wang 2021; Z. Wang et al. 2014).

BOLD time‐series with high entropy indicates the signal is less predictable or more random, associated with a greater capacity for processing information (Wehrheim et al. 2024). On the contrary, low entropy indicates that the BOLD signal is more predictable or constant, linked to reduced brain complexity and inferior flexibility and efficiency in processing information (Niu et al. 2022; Saxe et al. 2018; Z. Wang et al. 2014).

There are multiple entropy measurements available to study biological systems. For instance, Pincus (1991) introduced approximate entropy (ApEn) to examine entropy through the complexity of a signal (Pincus 1991). Nevertheless, due to its high sensitivity to sample size and its low consistency, Richman and Moorman (2000) introduced sample entropy (SampEn) (Richman and Moorman 2000). The SampEn analysis was developed with relative independence from the sample size, requiring less computational power than ApEn and showing higher consistency across measurements. Afterward, multiscale entropy (MSE) was introduced to account for multiple temporal scales in physiological data by considering complex temporal fluctuations (Costa et al. 2002, 2005).

Each entropy measurement has its own specific calculation; nevertheless, they are related. SampEn is a slightly modified version of ApEn (Richman and Moorman 2000), and MSE is a generalized version of SampEn (Costa et al. 2005). The mathematical formulae presented here focus on SampEn. For SampEn, in addition to standard rsfMRI preprocessing (Section 2 Standard Preprocessing for rsfMRI Data), a high‐pass temporal filtering (0.01 Hz) is typically applied (Saxe et al. 2018; Sokunbi et al. 2013; Z. Wang et al. 2014; Xue et al. 2019; Zhou et al. 2016).

Entropy is a local brain activity (i.e., voxel‐wise) method computed as the average uncertainty of a time series, measured by computing the negative logarithm of the conditional probability that two vectors remain similar at the next point, excluding self‐matches (Richman and Moorman 2000; Sokunbi et al. 2013). For a BOLD time‐series signal consisting of *N* time points, denoted as $X\left(t\right)=\left(x_{t=1},x_{t=2},\ldots ,x_{t=N}\right)$, the first step is to form a series of vectors extracted from *X*(*t*) of length *m* denoted as $u_{m}\left(i\right)$:

$$u_{m}\left(i\right)=\left[x_{i},x_{i+1},\ldots ,x_{i+m-1}\right],i\in \left[1,N-m+1\right].$$

Let $n_{i}^{m}\left(r\right)$ be the number of vectors $u_{m}\left(j\right)$ that are close in distance to $u_{m}\left(i\right)$, by a tolerance of *r*:

$$n_{i}^{m}\left(r\right)=\sum_{j=1,j\neq i}^{N-m}d\left[u_{m}\left(i\right),u_{m}\left(j\right)\right]\leq r,\text{where}\;d:\text{distance measurement}.$$

Then, the probability that $u_{m}\left(j\right)$ is close to $u_{m}\left(i\right)$ is defined as

$$C_{i}^{m}\left(r\right)=\frac{n_{i}^{m}\left(r\right)}{N-m+1}.$$

The average of $C_{i}^{m}\left(r\right)$ represents the probability that any two vectors are within a tolerance distance *r*:

$$C_{i}^{m}=\frac{1}{N-m}\sum_{i=1}^{N-m}C_{i}^{m}\left(r\right).$$

With this approach, the BOLD signal is segmented into smaller sets of size *m*, and the distance for each segment is compared against the rest of the segments. If the Euclidean distance is smaller than the threshold *r*, then the vectors are “close enough.” This process is then repeated for sets of the BOLD signal of size *m* + 1. SampEn is computed as

$$\text{SampEn}\left(N,r,m\right)=-\ln\left[\frac{C_{i}^{m+1}}{C_{i}^{m}}\right],$$

where

- N is the number of time points in the time series.
- m is the BOLD segmentation set length.
- r is the distance tolerance value.

An entropy value of 0 would mean that $C_{i}^{m}=C_{i}^{m+1}$, that is, the BOLD signal is fully predictable. The typical parameters used for rsfMRI analysis are m=3 and r=0.6, as simulations indicated these parameters to be optimal (Saxe et al. 2018; Z. Wang et al. 2014; Xue et al. 2019).

## 4 Discussion

**FIGURE 2:**
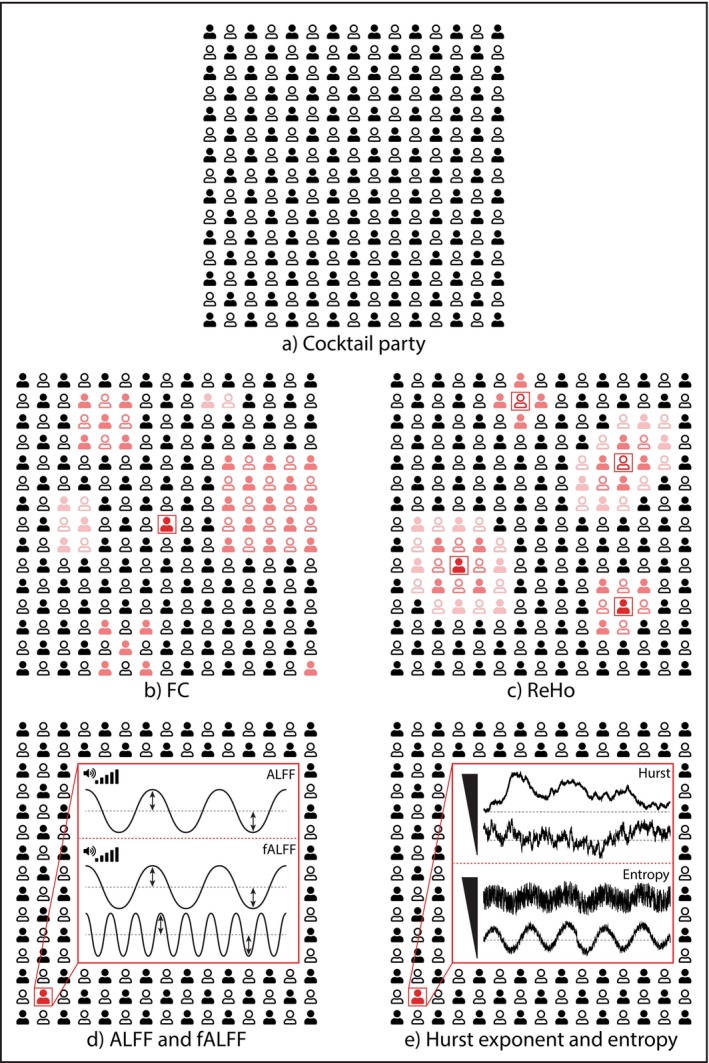
(a) Cocktail party analogy, where everyone records their talks through the night. (b) FC represents people in the room talking similarly to a specific person shown in the red square. (c) ReHo measures how similar a radius of people are talking to the person at the center. ReHo is computed for each person in the room. The red color scale represents the strength of similarity. (d) ALFF depicts how loud each person is talking in a low pitch, while fALFF normalizes this measurement by the entire frequency range of the conversation. (e) H represents how random or consistent each person is speaking. Lastly, entropy shows how regular and predictable or spontaneous and expressive each person speaks.

This narrative review provides an overview of rsfMRI foundations, general preprocessing procedures, and commonly used rsfMRI analyses. Furthermore, for each analysis, the theoretical framework, mathematical foundation, critical significance, and specific data preprocessing steps were described. Some of the most common rsfMRI analyses were outlined, each measuring a specific feature of the BOLD signal and corresponding physiological mechanism. An analogy that can be used to understand the features measured in the BOLD signal by each technique is shown in Figure 2a. Picture a cocktail party where each guest has a microphone to record their own conversation through the evening (i.e., the BOLD signal from each voxel within the brain). The rsfMRI techniques described can be thought of as approaches that characterize different properties of the recordings. FC looks at people around the room who are talking similarly compared to a specific person (i.e., a seed) (Figure 2b). ReHo represents how similar a radius of people (i.e., close neighbors) are talking to the person at the center, for each person within the room (Figure 2c). ALFF measures how loud each person is speaking with low pitch (i.e., LFF), while fALFF normalizes this measurement by how loud each person is speaking at all frequencies (Figure 2d). H depicts how erratic and unstable (H < 0.5) or smooth, rhythmic, and structured (H > 0.5) each person is speaking (Figure 2e). Lastly, entropy shows how regular and predictable (low entropy) or spontaneous and expressive (high entropy) each person speaks (Figure 2e).

Each rsfMRI metric offers distinct insights of the BOLD signal but carries important limitations that researchers should consider when selecting an analysis. FC is highly sensitive to seed selection (seed‐based), motion, physiological noise, and preprocessing choices (Cole et al. 2010; Griffanti et al. 2014; Power et al. 2012). ALFF and fALFF are influenced by physiological noise, with fALFF improving specificity (Jia et al. 2020; Zou et al. 2008). To note that studies diverge in the choice of frequency band, with some authors using 0.01–0.08 Hz (Chao‐Gan and Yu‐Feng 2010; Whitfield‐Gabrieli and Nieto‐Castanon 2012; Zang et al. 2007; Zou et al. 2008) while more recent work employs 0.01–0.1 Hz (Birn 2023; C. G. Yan, Craddock, et al. 2013). ReHo captures only local synchrony, with results dependent on neighborhood size and smoothing parameters (C. G. Yan, Cheung, et al. 2013; Zang et al. 2004). H estimates can vary by calculation method and scan length and may reflect vascular or nonneural sources alongside scale‐free neural dynamics (Campbell and Weber 2022; Eke, Herman, Sanganahalli, et al. 2012). Entropy measures are highly dependent on parameter choices, data length, and calculation method (Sokunbi 2014; Z. Wang et al. 2014; A. C. Yang et al. 2018). Together, these limitations highlight the need for careful methodological choices and cautious interpretation when applying each approach.

**FIGURE 3:**
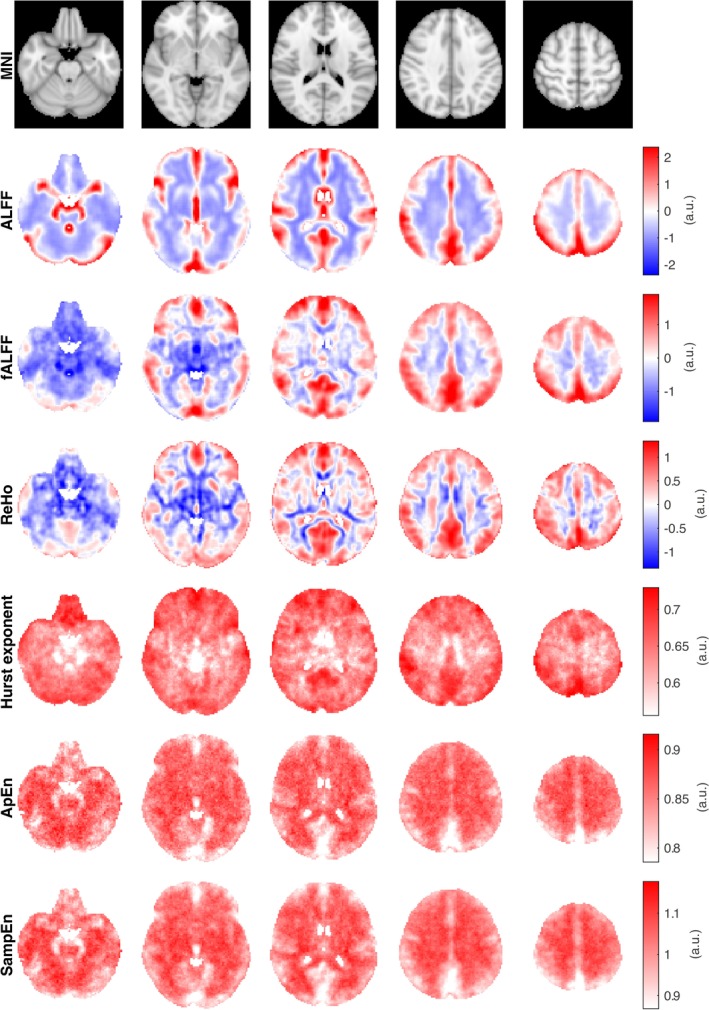
Plot showing a voxel‐wise average of each rsfMRI analysis. Five brain slices are shown, where the colormap represents the output value for each analysis. It is noted that ALFF, fALFF, and ReHo maps are normalized by subtracting the mean and dividing by the standard deviation. rsfMRI datasets from 14 healthy participants (50:50 sex ratio, 18–22 years old) were retrieved from INDI (available at https://www.nitrc.org/projects/fcon_1000/), from the Beijing Normal University, State Key Laboratory of Cognitive Neuroscience and Learning Enhanced Sample. Each rsfMRI analysis was applied to each dataset, producing 14 output maps per analysis. Subsequently, the mean voxel‐wise value was computed for each rsfMRI analysis.

**FIGURE 4:**
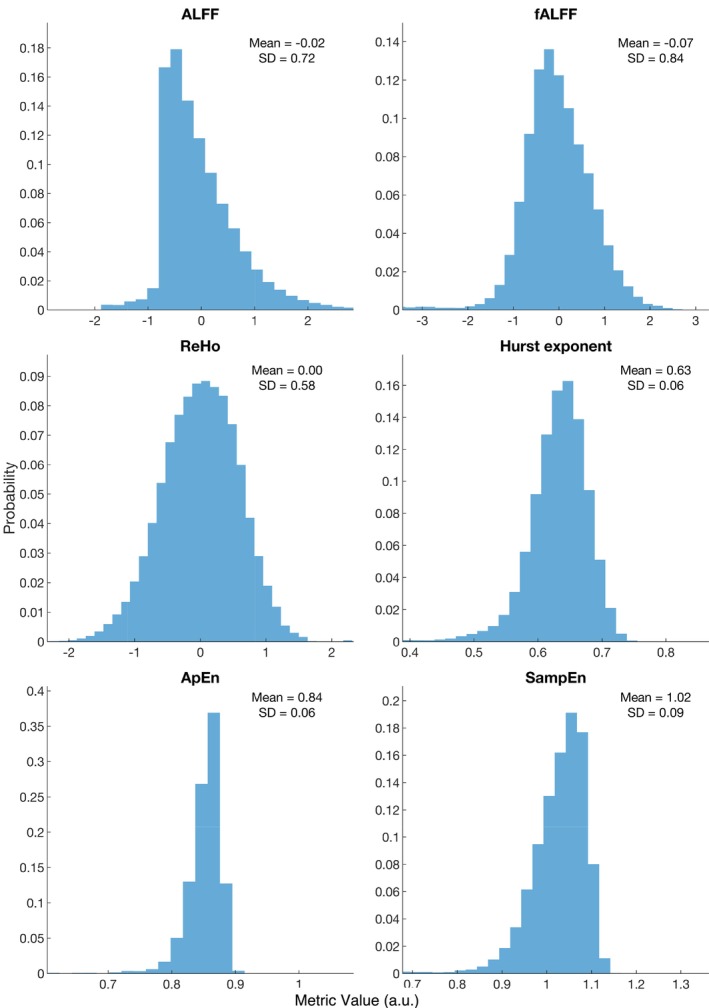
Plot showing the distribution of voxel‐wise averages of each rsfMRI analysis. The histograms show whole‐brain output values for each analysis. These histograms show the dynamic range, mean, and standard deviation of each analysis. To note that ALFF, fALFF, and ReHo maps are normalized by subtracting the mean and dividing by the standard deviation. rsfMRI datasets from 14 healthy participants (50:50 sex ratio, 18–22 years old) were retrieved from INDI (available at https://www.nitrc.org/projects/fcon_1000/), from the Beijing Normal University, State Key Laboratory of Cognitive Neuroscience and Learning Enhanced Sample.

All analyses discussed here can be understood as functions with an input and output. The input is equivalent across all rsfMRI analyses: the preprocessed BOLD signal. Nonetheless, each analysis uses a different analytical procedure, thus yielding a distinct feature of the BOLD signal. While these differences may not be immediately apparent in the conceptual and mathematical overview, they become clear when visualized in a spatial colormap, as shown in Figure 3. This figure shows the averaged output of each analysis (i.e., the distribution and intensity of the output values) using the same rsfMRI datasets from 14 healthy participants as the input for all computations (50:50 sex ratio, 18–22 years old) retrieved from the open‐access International Neuroimaging Data‐Sharing Initiative (INDI) (available at https://www.nitrc.org/projects/fcon_1000/). It is noted that each analysis depicted a different spatial distribution and intensity across brain regions, where each metric showed a particular contrast between regions. To illustrate, ALFF and fALFF maps showed higher values in regions closer to the cerebral cortex, while there were lower values in subcortical areas, consistent with previous studies (H. Yang et al. 2007; Zuo et al. 2010). A similar trend between the cerebral cortex and subcortical regions has also been identified in studies examining H (Campbell et al. 2021; Campbell and Weber 2022). Moreover, the distribution of values for each analysis is different, where each one shows a specific dynamic range. This is clear in the whole‐brain histograms for each analysis (Figure 4), where ApEn, SampEn, and H display a tighter distribution than ALFF, fALFF, and ReHo. Furthermore, the link between entropy measurements is seen in spatial maps, where ApEn and SampEn show similar patterns yet different dynamic ranges (Richman and Moorman 2000).

It is widely recognized that the selection of preprocessing parameters impacts rsfMRI analyses and statistics (Liang et al. 2012; Specht 2019; J. Yang et al. 2020). Here, we show that not only does preprocessing impact the results, but so does the choice of rsfMRI analysis. While these observations hold significance, they should be interpreted cautiously, as they do not reflect the overall normative distribution of each rsfMRI analysis. With the increasing growth of big data and publicly available large brain datasets (Poldrack and Gorgolewski 2014; Xia and He 2017; Yamashita et al. 2019), there is an opportunity to establish a multivariate normative and diseased distribution with these rsfMRI analyses. This can lead to new research questions, enhance generalizability, and increase the reliability and accuracy of rsfMRI findings (Menagadevi et al. 2023; Poldrack and Gorgolewski 2014; Xia and He 2017; J. Yang et al. 2020). Further, as more analyses are developed or adopted for rsfMRI, every new analysis should be challenged and compared to the existing ones to better understand the rsfMRI representation of physiologically healthy and nonhealthy brain states.

Even at rest, the brain is performing dynamic, complex activities (Damoiseaux et al. 2006; Rabinovich et al. 2012). As such, the BOLD signal shows short‐scale spatiotemporal variations (Allen et al. 2014; Thompson 2018), which means it has nonstationary statistical properties that change over time (Perraudin and Vandergheynst 2017). Techniques such as FC may miss details of these variations as the correlation coefficient is computed across the entire span of the rsfMRI scan. In the past decade, there has been an increase in proposed time‐varying and dynamic rsfMRI approaches (Calhoun, Miller, et al. 2014; Hutchison et al. 2013; Iraji, Faghiri, et al. 2021; Iraji, Miller, et al. 2020; Preti et al. 2017), such as sliding‐window analyses (Allen et al. 2014; Handwerker et al. 2012), wavelets (Chang and Glover 2010), and dynamic ICA (Bravo Balsa et al. 2024; H. Zhang et al. 2022). Studying these spatiotemporal variations in the BOLD signal may provide new insights into brain functioning and lead to a more comprehensive understanding of the healthy brain and more disease biomarkers.

The analyses outlined in this review describe different measurement characteristics of the BOLD signal, such as its power, complexity, and local or global connectivity. Although each analysis could detect brain irregularities in diseased populations, combining analyses could provide a more detailed description of brain function and impairment, especially in the context of complementary analyses. The use of multiple rsfMRI analyses could enhance sensitivity to brain activity, improve the detection of brain abnormalities, and provide a joined description of temporal and spatial characteristics of brain function (de Vos et al. 2018; Meier et al. 2017; Z. Zhang 2024).

## 5 Limitations and Future Directions

Although extensive, this review does not encompass all the available rsfMRI analyses. Other metrics, such as effective connectivity (dynamic causal modeling or Granger causality) (Seth et al. 2015; Sharaev et al. 2016), connectome gradients (Margulies, Ghosh, et al. 2016), multivariate pattern analyses (Nieto‐Castanon 2022), brain avalanche (L. Xu et al. 2022), Lempel–Ziv complexity (Shumbayawonda et al. 2018), and Lyapunov exponent (LE) (Wolf et al. 1985), among others, were not considered because they are more complex approaches, thus falling outside of the scope of this review. For instance, the LE is an analysis utilized to characterize deterministic chaos in nonlinear dynamic systems (Wolf et al. 1985). It allows the visualization of the dynamics of a time domain signal, revealing patterns that otherwise would not be apparent (Keilholz et al. 2020; Kutepov et al. 2020; Valenza et al. 2012). Furthermore, the BOLD signal is fundamentally a time domain signal, reflecting the neurovascular coupling over the sequence acquisition time. Therefore, other analyses used to study neurological signals, such as electroencephalography (EEG) or magnetoencephalography (MEG), could be used comparatively to study additional BOLD signal properties, expanding the range of available rsfMRI analysis tools.

Typically, rsfMRI studies acquire the whole brain within a 2‐s temporal resolution (i.e., 0.5 Hz) (Bollmann and Barth 2021; Raimondo et al. 2021). However, this relatively low sampling rate limits the highest frequency that can be studied to 0.25 Hz (i.e., the Nyquist limit), which reduces the ability to detect intrinsic noise and physiological artifacts (Cahart et al. 2023). Multiband (MB) imaging has recently emerged as a popular method to speed up the acquisition of rsfMRI scans (Larkman et al. 2001; Risk et al. 2021). MB simultaneously acquires multiple slices, reducing the time needed to scan the entire brain to typical times of 500 ms or four times faster than previously done (Cahart et al. 2023; Mark et al. 2021; Raimondo et al. 2021; J. Xu et al. 2013). Thus, MB enables the collection of more time points, yielding higher temporal resolution (Cahart et al. 2023; Risk et al. 2021). With the increased adoption of MB in rsfMRI, the reliability of these rsfMRI metrics could improve. For instance, it has been shown that reproducibility and reliability increase with higher sampling rates for FC (Golestani et al. 2017; Liao et al. 2013; X. Wang et al. 2013), ReHo (Golestani et al. 2017; Q. H. Zuo et al. 2013), and H (Dona, Hall, et al. 2017; Eke, Herman, Bassingthwaighte, et al. 2000). Further research is needed to investigate the effects of increased temporal resolution in rsfMRI metrics, as higher sampling rates could allow for better characterization of spurious signals and respiratory and cardiac noise.

Lastly, although comprehensive, this review does not follow the formalism of a systematic review. Given the variability in clinical populations and their characteristics across techniques, a systematic review was not feasible, nor was it our goal. Our goal was to provide a structured “dictionary” of rsfMRI analyses rather than a prescriptive guide on their application to specific research domains or clinical populations. Future research could focus on specific diseases and populations to allow rigorous comparisons across techniques.

## 6 Conclusion

This narrative review presents commonly used and clinically germane rsfMRI metrics and analyses to examine several features of the BOLD signal that can be used to understand the brain and characterize subtle alterations. This review comprehensively describes five rsfMRI analyses, covering conceptual framework, mathematical foundation, and overall significance. It is crucial to apply rsfMRI analyses that most accurately answer specific questions because each analysis will exhibit different BOLD signal features having distinct interpretations, spatial distributions, intensities, and contrasts across brain regions. This review aimed to provide a conceptual, mathematical, and practical overview of key measures, which, when complemented with their subject matter expertise, can lead to appropriate rsfMRI metric selection.

rsfMRI is an increasingly valuable tool for studying brain function in both healthy and disease conditions. As such, researchers and clinicians should be aware of which rsfMRI analysis to apply, a decision that strongly depends on the research question and the BOLD signal properties that are relevant to answering it. Rather than selecting the most popular tool, the a priori decision‐making of which rsfMRI analysis to use could provide a better fit between the research question and the outcome variables. This could further expand the knowledge of brain health and function, which, once firmly established, will increase the chances of finding clinically relevant biomarkers in diseased populations.

## Author Contributions

**Alejandro Amador‐Tejada:** data curation, formal analysis, methodology, software, validation, visualization, writing – original draft, writing – review and editing. **Bhanu Sharma:** data curation, formal analysis, writing – original draft, writing – review and editing. **Ethan Danielli:** data curation, formal analysis, writing – original draft, writing – review and editing. **Michael D. Noseworthy:** conceptualization, funding acquisition, project administration, resources, supervision, writing – original draft, writing – review and editing.

## Conflicts of Interest

Dr. Noseworthy is the co‐founder and CEO of TBIFinder Inc., a data analytics company that focuses on brain injury. Also, Dr. Danielli and Mr. Amador‐Tejada are part‐time research interns with TBIFinder Inc. There is no overlap between TBIFinder and the current research presented in this review.

## Peer Review

The peer review history for this article is available at https://www.webofscience.com/api/gateway/wos/peer‐review/10.1111/ejn.70276.

## Data Availability

All data analyzed in this work was sourced from the International Neuroimaging Data‐Sharing Initiative (INDI) (available at https://www.nitrc.org/projects/fcon_1000/). We would gladly share which exact brains we analyzed.
